# Mechanisms Governing the Stability of Fe-As Complexes: Roles of Environmental and Material Intrinsic Factors

**DOI:** 10.3390/toxics14010104

**Published:** 2026-01-22

**Authors:** Zhonglan Yang, Tianlai Ouyang, Shiming Su, Yanan Wang, Fengxian Yao, Zhiqiang Ding, Mengmeng Yan, Xibai Zeng

**Affiliations:** 1 National Navel Orange Engineering Research Center, School of Life Sciences, Gannan Normal University, Ganzhou 341000, China; zhonglanyang@163.com (Z.Y.); fengxianyao@aliyun.com (F.Y.);; 2 Institute of Agricultural Environment and Sustainable Development, Chinese Academy of Agriculture Sciences, Beijing 100081, China

**Keywords:** arsenic immobilization, Fe-As complexes, ferrihydrite aging, competitive anions, contaminated site remediation

## Abstract

Arsenic (As) contamination threatens ecosystems and human health, and iron (hydr)oxides-mediated formation of Fe-As composites is a key strategy for arsenic immobilization, while the long-term stability of these composites under complex environmental conditions remains a critical concern. This study systematically investigated the interactive effects of environmental factors (temperature: 5–35 °C, pH: 4–8, competing ions: phosphate and citrate) and material intrinsic properties (ferrihydrite aging: 0–60 days, Fe/As molar ratio: 1.875 and 5.66, adsorption time) on Fe-As composite stability using multiscale characterization techniques and theoretical modeling. Results showed that temperature was the dominant controlling factor, with arsenic release increasing by 4.25% per 1 °C rise (178% higher at 35 °C vs. 20 °C) and an exponential relationship model established (R^2^ = 0.96). Ferrihydrite aging enhanced stability, as 60-day aged composites (Fh_60d_-As) exhibited minimal arsenic release (18.83%) at pH 4/20 °C, attributed to increase As(V)-O-Fe binding energy (1.2 eV) and -OH group enhancement (12.5%). Phosphate induced 2.4-fold higher arsenic release than citrate, and lower pH (4–6) reduced release via enhanced protonation. A stability prediction model was developed (R^2^ = 0.91), and practical remediation strategies were proposed: maintaining temperatures below 25 °C in arsenic-containing waste repositories and using pre-aged iron-based materials. This work provides quantitative benchmarks and mechanistic insights for contaminated site rehabilitation.

## 1. Introduction

Arsenic (As), a toxic metalloid ubiquitous in soil and water environments, poses severe and persistent threats to ecological integrity and human health globally [1]. The World Health Organization (WHO) has set a strict safe limit of 10 μg/L for arsenic in drinking water, yet millions of people worldwide remain exposed to excessive concentrations due to natural geogenic processes and anthropogenic activities such as mining, smelting, and agricultural chemical use [2]. Recent epidemiological evidence underscores the urgency of this issue; even low-to-moderate arsenic exposure (<100 μg/L) is positively associated with increased risks of stroke, ischemic heart disease, and acute myocardial infarction, with more pronounced effects observed in men [3]. In coastal regions of Bangladesh, the interaction of arsenic with salinity further exacerbates health risks, leading to higher morbidity rates of water-related diseases and cardiovascular disorders through cooking and washing water exposure [4]. In China, 2024 surveys show that residents in high-arsenic drinking water areas (e.g., Guizhou, Inner Mongolia) have a urine arsenic median of 86 μg/L—over 8 times the WHO threshold—and a skin keratosis prevalence 4.3 times higher than non-exposed populations [5].

In aerobic environments, arsenate (As(V))—the predominant and highly mobile form of arsenic—can be effectively immobilized by iron (hydr)oxides through adsorption and coprecipitation, forming iron-arsenic (Fe-As) composites [6,7]. This approach remains the dominant remediation strategy globally, with iron-based materials accounting for most large-scale arsenic pollution control projects [5,8]. However, the long-term stability of these Fe-As composites is the cornerstone of successful remediation, as their destabilization can lead to arsenic remobilization and recontamination [9]. Emerging advanced remediation materials have shown enhanced performance in As(III) oxidation-adsorption or multi-pollutant reusable removal [10,11], yet iron-based materials retain irreplaceable advantages for large-scale applications due to their cost-effectiveness [12].

Numerous studies have explored factors influencing Fe-As composite stability, focusing on material intrinsic properties and external environmental conditions [6]. Among the intrinsic factors, the Fe/As molar ratio plays a key role: lower ratios intensify competition between arsenic and coexisting anions for adsorption sites, increasing desorption risk [13,14]. The aging process of ferrihydrite—a common iron (hydr)oxide used in remediation—has sparked contradictory findings: some studies report reduced stability due to decreased specific surface area [15], while others observe enhanced stability from arsenic incorporation into the iron oxide lattice [16]. Recent molecular-level research reveals that hematite, a product of ferrihydrite aging, exhibits facet-dependent selectivity for arsenate over phosphate: the (001) facet forms more stable inner-sphere complexes via dual iron site interactions, offering new insights into aging-induced stabilization mechanisms [17,18,19]. Additionally, prolonged adsorption time drives transformation from metastable to kinetically stable structures [20].

External environmental factors, including pH, temperature, and competing ions, also exert significant impacts [21,22]. pH regulates the protonation state of arsenate and iron oxide surface charge, modifying electrostatic interactions [23]. Temperature changes alter iron oxide recrystallization dynamics and arsenate activation energy, accelerating release [24,25]. Competing ions like phosphate and citrate complicate stability: phosphate outcompetes arsenic through a similar tetrahedral structure and inner-sphere complexation [26], while citrate induces iron oxide dissolution [27]. A study on hematite facets further clarifies that arsenate selectivity is enhanced at higher concentrations and varies with exposed crystal planes, highlighting the complexity of anion competition in real environmental matrices [28,29].

Despite these advances, most previous research has focused on single-factor effects, neglecting the complex interactions between material properties and environmental conditions that govern Fe-As composite stability in real-world scenarios [30]. This gap persists even as global arsenic governance demand grows—China alone faces hundreds of millions of tons of legacy arsenic slag, with the market for arsenic pollution control projected to exceed CNY 15 billion by 2030 [31,32]. The lack of systematic understanding of multi-factor interactive mechanisms hinders the optimization of remediation strategies for long-term reliability [33]. To address this, the present study investigates Fe-As composite stability under combined intrinsic (ferrihydrite aging time, Fe/As molar ratio, adsorption time) and external (pH, temperature, competing ions) factors. Using multi-scale characterization techniques and theoretical modeling, we aim to identify key controlling factors, clarify stabilization mechanisms, and develop practical remediation guidelines. This study provides quantitative benchmarks and mechanistic insights for predicting long-term Fe-As composite stability, offering valuable guidance for the design of robust arsenic remediation strategies in contaminated sites.

## 2. Materials and Methods

### 2.1. Synthesis and Aging Treatment of Fe-As Complexes

Ferrihydrite (Fh) was synthesized at 25 °C according to the method in the literature [34]. Three grams of Fh powder was resuspended in 0.01 mol L^−1^ solution of sodium nitrate in 50 mL polyethylene bottles to reach a final Fh concentration of ~0.67 mol Fe L^−1^. Subsequently, to obtain the preaged Fh, the suspension was aged for 10 d (Fh_10d_), 30 d (Fh_30d_) and 60 d (Fh_60d_) in a water bath at 75 °C. To distinguish between ferrihydrite samples, the Fh sample without preaging is termed Fh_0d_ in the remaining text.

Sodium arsenate (Na_3_AsO_4_ 12H_2_O, ≥99%) was mixed with 45 mL of Fh_0d_, Fh_10d_, Fh_30d_, and Fh_60d_ to reach Fe/As molar ratios of 1.875 and 5.66 (R1.875 and R5.66). Subsequently, the suspension containing arsenic was cultured in a water bath at 75 °C for 1 h and 240 d to obtain Fe-As complexes with different Fe/As molar ratios (R1.875 and R5.66) and adsorption times (Fh-As_1h_, Fh_10d_-As_1h_, Fh_30d_-As_1h_, Fh_60d_-As_1h_, Fh-As_240d_, Fh_10d_-As_240d_, Fh_30d_-As_240_ and Fh_60d_-As_240d_). The samples were centrifuged (5000 rpm for 10 min) and washed three times using sodium nitrate. The solid phase was freeze-dried, ground into fine powder, and stored at −20 °C for composition analysis and sample characterization. Fe-As complexes were dissolved using an aqua regia solution and filtered through 450 nm membranes to obtain supernatants for measuring the concentrations of total arsenic, ensuring the accuracy of the following tests for the As loading rate.

### 2.2. Stabilization Test of Fe-As Complexes

In this study, based on the results of a previous study [19], representative anions in the environment that can compete with arsenate for adsorption, phosphate (PO_4_, Na_3_PO_4_), and citrate (CIT, Na_3_C_6_H_5_O_7_), were used to test the stability of Fe-As complexes.

Ten grams of Fe-As complexes synthesized from 2.1 with different aging times, Fe/As molar ratios and adsorption times were placed in centrifuge tubes, and the pH of the system was adjusted to 4, 6, and 8 after the addition of competing ions (molar ratio of competing ions to As(V) of 1), with a final solid/liquid ratio of 1:100 [20] and three replicates. The samples were shaken for 24 h at room temperature (20 ± 2 °C) in the dark [33].

The same Fe-As complexes were also taken separately in centrifuge tubes after the addition of competing anions (molar ratio of competing ions to As(V) of 1), and the pH of the system was adjusted to 6 for a final solid/liquid ratio of 1:100 [33]. The samples were shaken for 24 h at 5, 20 and 35 °C in the dark, and three replicates were performed. The experiment was carried out by adjusting the pH with 0.1 mol·L^−1^ HCl and NaOH every hour for the first 8 h and every 4 h thereafter. After the 24 h shaking period, the final pH of the supernatant was measured. The values remained stable at 3.9 ± 0.2, 6.0 ± 0.2, and 8.1 ± 0.3 for the target pH of 4.0, 6.0, and 8.0, respectively, confirming that the experimental conditions were well maintained and that the observed effects are representative of the intended pH levels.

The samples obtained were centrifuged (5000 rpm, 10 min), and the supernatant was passed through a 450 nm filter membrane for measurement. The total arsenic was determined by hydride generation atomic fluorescence spectrometry (HG-AFS) (Jitian, AFS-9120, Beijing, China). The solids were washed, freeze-dried, ground and stored at −20 °C for composition analysis and sample characterization.

### 2.3. Characterization of the Fe-As Complexes Before and After Leaching

The composition of the solid phases was characterized by an X-ray diffraction (XRD) (Rigaku, Ultima IV, Tokyo, Japan) with Cu Kɑ radiation (λ = 0.1541 nm) at 40 kV with a 40 mA current. The scan range was between 10° and 75°, with a step size of 0.02° and duration of 1 s step^−1^. Phase identification was performed by matching XRD patterns with the standard reference card for hematite (JCPDS No. 33-0664), which clearly delineates the characteristic diffraction angles of the (104) and (110) crystal planes. XRD patterns were analyzed using JADE 6.5 software (Materials Data Inc., Silicon Valley, CA, USA) to identify the formation of new phases. The infrared spectra of each sample after competition were collected using a Fourier transform infrared spectroscopy (FTIR) spectrometer (Thermo Fisher, Nicolet 6700, Waltham, MA, USA) in the 400–4000 cm^−1^ range at 0.09 cm^−1^ measurement resolution. The different states of oxygen in Fe-As complexes were determined by an X-ray photoelectron spectrometer (XPS) (Shimadzu Co., Amicus Model, Kyoto, Japan) with monochromatic Al Kα radiation (1486.8 eV). The morphology and elemental composition of the selected minerals were investigated by scanning electron microscopy-energy dispersive X-ray spectroscopy (SEM-EDS) using instruments from Hitachi SU8020 (Tokyo, Japan) and HORIBA EX250 (Kyoto, Japan).

## 3. Results

### 3.1. Effect of the Fe/As Molar Ratio and Aging on the Stability of Complexes

**Figure 1:**
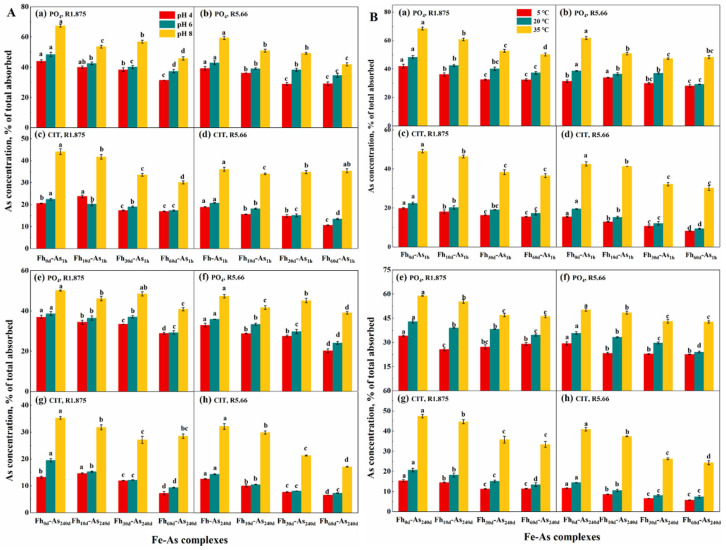
Under the interaction of pH (4, 6 and 8) and ferrihydrite aging (aging 0, 10, 30 and 60 d) at 20 °C (**A**) or under the interaction of temperature (5, 20 and 35 °C) and ferrihydrite aging (aging 0, 10, 30 and 60 d) at a pH of 6 (**B**), the As(V) content of phosphate or citrate extracted from Fe-As_1h_ (**a**–**d**) and Fe-As_240d_ (**e**–**h**) complexes (initial Fe-As molar ratios of 1.875 and 5.66) as a proportion of the total arsenic adsorbed from Fe-As complexes.

As the Fe/As molar ratio decreases, the amount of co-sided FeO_6_ octahedra of iron oxides is subsequently reduced or even absent, i.e., the free sites on the surface of the complexes are reduced, enhancing the competition between competing ions and arsenic and thus reducing the stability of the complexes. Therefore, the arsenic content leached from the complexes at R1.875 (42.23%) > R5.66 (36.92%) (Figure 1).

The stability of the complexes increased as the aging time of the ferrihydrite increased, i.e., the average arsenic contents of Fh-As, Fh_10d_-As, Fh_30d_-As and Fh_60d_-As leached by PO_4_ were 45.15%, 40.15%, 37.29% and 35.38%, respectively. When subjected to various environmental factors, the average arsenic contents released from the Fe-As complexes formed by adsorbed arsenate after 0 d, 10 d, 30 d and 60 d of ferrihydrite aging were 35.27%, 31.68%, 28.63%, and 25.80%, respectively. The release rate of arsenic from the complexes decreased with increasing aging time and followed the following pattern: within 10 d of aging, the release of arsenic decreased by 0.36% for each additional day; within 10 d–30 d of aging, the release of arsenic decreased by 0.15% for each additional day; within 30 d–60 d of aging, the release of arsenic decreased by 0.09% for each additional day; and within 60 d of aging, the release of arsenic decreased by 0.16% for each additional day of aging (Figure 1).

### 3.2. Changes in Arsenate Stability During the Adsorption of Iron Minerals with Arsenic

**Figure 2:**
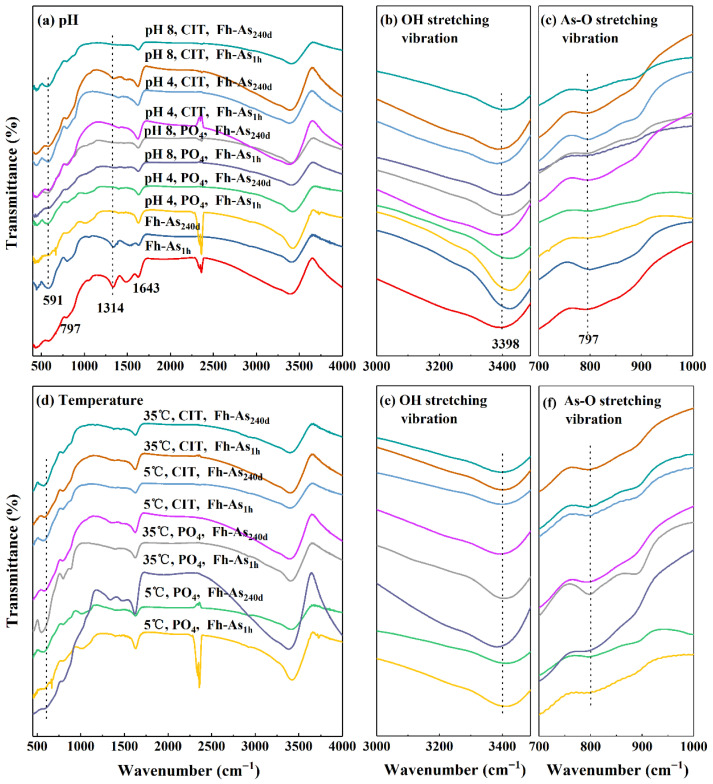
FTIR scanning spectra of Fe-As complexes before and after extraction by PO_4_ and CIT at pH values of 4, 6, and 8 (**a**) or at 5 °C, 20 °C, and 35 °C (**d**). FTIR spectra in the range of 3000–3600 cm^−1^ corresponding to the OH stretching vibrations of Fe-As complexes before and after extraction by PO_4_ and CIT at pH values of 4, 6, and 8 (**b**) or 5 °C, 20 °C, and 35 °C (**e**). FTIR spectra in the range of 700–1000 cm^−1^ corresponding to the As-O bond stretching vibrations of Fe-As complexes before and after extraction by PO_4_ and CIT at pH values of 4, 6, and 8 (**c**) or 5 °C, 20 °C, and 35 °C (**f**).

As the adsorption time of arsenate and iron oxides increased, the stability of the complexes increased, i.e., the average arsenic content leached by PO_4_ from Fh-As_240d_ (31.61%) was lower than that of Fh-As_1h_ (37.93%) (Figure 1). The stronger the hydrogen bond formed between the anions in the crystal and the structural H_2_O, the lower the wavenumber of the H_2_O-OH stretching vibration peak (FITR 3000–3600 cm^−1^) [9]. From Figure 2b, the Fe-As complexes all show a shoulder peak on the lower side of the main peak at 3398 cm^−1^, indicating that the ion interacts with the structured water via H-bonding and that the OH moves to higher energy as the adsorption time increases.

**Figure 3:**
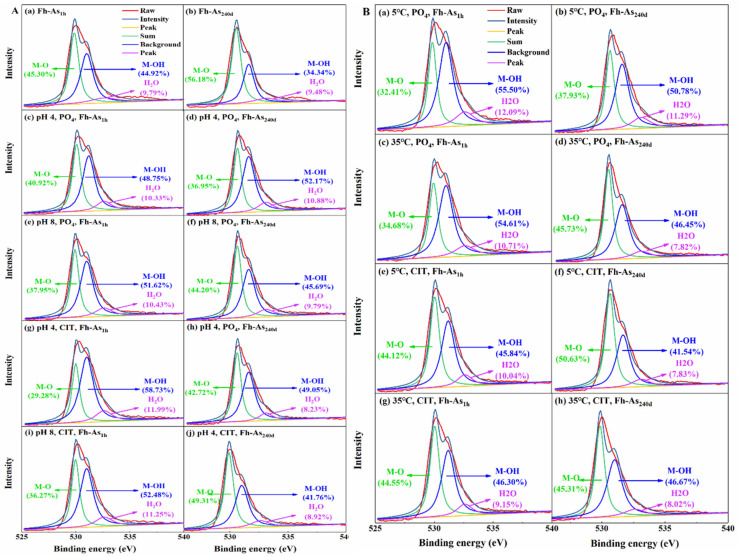
XPS O 1s scanning spectra of Fe-As complexes before and after extraction by PO_4_ and CIT at pH values of 4, 6 and 8 (**A**) or 5 °C, 20 °C and 35 °C (**B**).

The binding of arsenate to iron oxides goes beyond surface adsorption and results in the formation of more stable inner-sphere Fe-As complexes, which is confirmed by the characteristic peak of the bound XPS As 3d moving toward higher wavenumbers after leaching (Figure S1). Additionally, with increasing reaction time, the shift in this peak increased, i.e., the shift of Fh-As_240d_ > Fh-As_1h_ (Figure S1b,e), confirming that Fh-As_240d_ forms more stable inner-sphere complexes. It is evident from the peak splitting results that the O 1s XPS spectrum can be divided into three peaks, M-O (Fe-O, As-O, 529.4–530.3 eV), M-OH (Fe-OH, As-OH, 530.6–531.4 eV) and adsorbed H_2_O (532.4–532.8 eV) [9]. As the reaction time increased from 1 h to 240 d, the Fe-O content increased from 45.3% to 56.18% (Figure 3A(a,b)), indicating that arsenic gradually entered the mineral lattice from the Fe oxide surface to form Fe-O-As; meanwhile, the Fe-OH content decreased from 44.92% to 34.34% and the content of crystalline H_2_O decreased from 9.79% to 9.48%.

The stability of the Fe-As complexes is enhanced with increasing adsorption time, which was more intuitively confirmed by SEM. Fh-As_1h_ consists of ~10 nm spherical particles connected flatly into a smooth surface block (Figure S2A), which is probably weakly crystalline iron arsenate [14]. In contrast, Fh-As_240d_ are 10 nm spherical particles aggregated into a rough, irregular microcrystalline agglomerate with a diameter of approximately 0.4 μm (Figure S2B), which may be scorodite [21]. The distributions of Fe, As, and O in Fh-As_240d_ are more tightly packed than those of Fh-As_1h_ (Figure S2B), mainly due to the gradual change in the bond from the single-dentate adsorption of Fh-As_1h_ to the double-dentate inner-sphere bond of Fh-As_240d_.

### 3.3. Role of the Reaction Media on the Stability of Complexes

The effect of PO_4_ on the stability of Fe-As complexes was greater than that of CIT, and the increase in arsenic leached by CIT with increasing pH or temperature was greater than that of PO_4_. Since PO_4_ and arsenate have similar structures and both form inner-sphere complexes with FeO, the arsenic content leached by PO_4_ from Fh-As_240d_ (36.04%) was higher than that of CIT (16.82%) (Figure 1). Since CIT induces dissolution of iron oxides to form 1–450 nm colloidal particles, which immobilize dissolved arsenic in the colloid again, it may also form binary or ternary complexes, showing a reduction in the ‘dissolved’ arsenic content; however, with changes in the environmental factors, the dissolution ability of CIT also changes, thus changing the ability of CIT to extract arsenic. This hypothesis is confirmed by the disappearance of the inward bending vibrational peak of the OH of the complex in the FITR spectrum (1314 cm^−1^) after leaching by PO_4_ and the presence of a weak peak intensity by CIT leaching (Figure 2). The intensity of the peak at 591 cm^−1^ in the FTIR spectrum (the telescopic vibrational peak of the OH structure of ferrihydrite) is significantly weaker after the complexes are leached by PO_4_ than by CIT (Figure 2d) and shifts toward lower binding energy. This may be due to the substitution of two OH or H_2_O on the FeO surface by PO_4_, i.e., the two O atoms of PO_4_ are coordinated with different Fe atoms to form Fe-O-P(O_2_)-O-Fe-type binuclear surface complexes. Additionally, PO_4_ forms an inner-sphere complex on the FeO surface, and the coordination structure leads to a reduction in its symmetry and a larger shift in the position of the absorption peak.

The leached arsenic content was higher by PO_4_ (39.58%) than by CIT (20.36%); however, with the change in pH, the arsenic content varied more by CIT than by PO_4_. The morphological differences between the CIT and PO_4_ leaches were significant. Fh-As_1h_ formed irregular microcrystalline particles with large pores by PO_4_ leaching, whereas by CIT leaching, the spherical particles aggregated into plate-like material, and as the pH increased, the pores of the bound material decreased and bonded more tightly into particles with smooth surfaces and larger particle sizes (Figure S5).

### 3.4. pH Dependency

**Figure 4:**
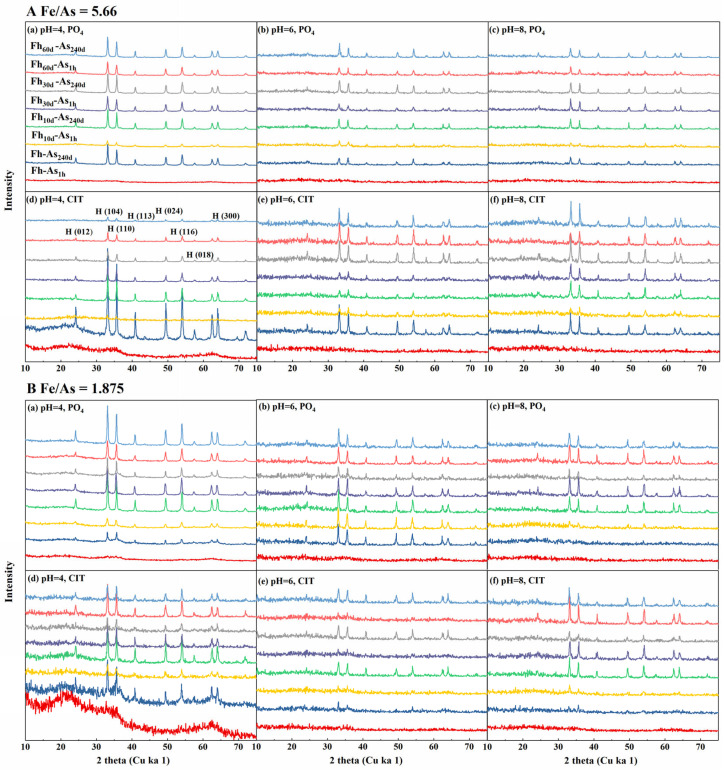
XRD pattern of Fe-As with Fe/As molar ratios of (**A**) R5.66 and (**B**) R1.875 before and after extraction by PO_4_ and CIT at pH values of 4, 6 and, 8, respectively. The newly formed substances are indicated in the graph as follows: H: hematite.

**Figure 5:**
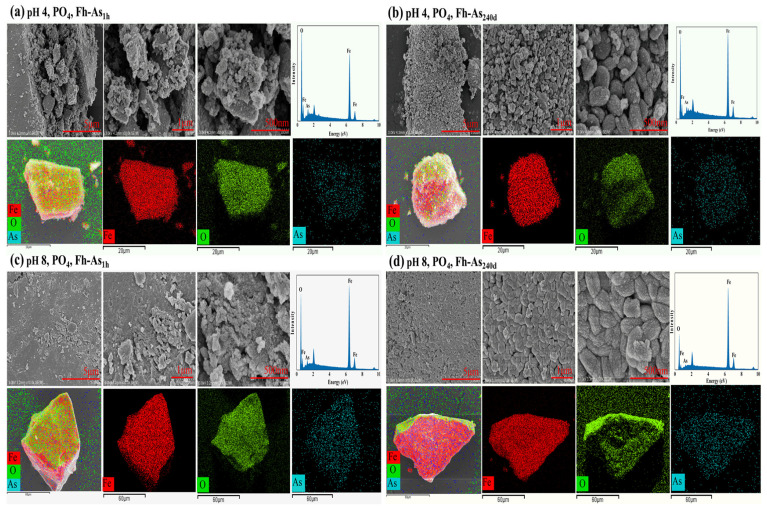
SEM images and EDS analysis of Fe-As complexes after extraction by PO_4_ from Fh-As_1h_ and Fh-As_240d_ at pH values of 4 and 8.

At 20 °C, the stability of the complexes decreased with increasing pH. At pH values of 4 and 6, PO_4_ leached 34.86% and 28.28% less arsenic from the Fh-As_1h_ complexes than at a pH of 8, respectively; CIT leached 53.34% and 49.19% less arsenic from Fh-As_1h_ than at a pH of 8 (Figure 1). It is possible that the release of arsenic was accelerated by the gradual change in the form of Fe^3+^ from Fe(OH)_2_^+^ to Fe(OH)_4_^−^ due to the increase in pH and the dissolution of iron oxides induced by CIT. Moreover, XRD showed that the crystallinity of the Fh-As complexes decreased with increasing pH, especially at a pH of 4, and the characteristic peaks of H(104) and H(110) in Fh-As_240d_ and Fh_60d_-As_240d_ after being leached by PO_4_ were significantly stronger than those at a pH of 8 (Figure 4A(a–c)). From the SEM, it was found that the complex material was extracted by PO_4_ at a pH of 4, the interparticle pores increased and the morphology was disrupted, changing into irregular aggregates, which was more significant in Fh-As_240d_ (Figure 5a,b).

The morphological disruption was more severe after the complexes were leached by PO_4_ at a pH of 8, changing from plate-like material to tightly bound irregular particles with reduced interparticle pore space (Figure 5c,d). In particular, Fh-As_240d_ aggregated from spherical particles into 0.6 μm oval particles that were tightly bound after leaching. Fh-As_1h_ complexes had larger pores prior to leaching, and under the influence of increasing pH, the minerals aggregated into irregular particles after leaching, whereas Fh-As_240d_ released less arsenic than Fh-As_1h_ and therefore aggregated into oval particles.

With increasing adsorption time, the minerals were more likely to form ellipsoidal particles after leaching (Figure S5), i.e., spherical particles aggregated into oval microcrystals of 250 nm and 400 nm after Fh-As_240d_ was leached by CIT at a pH values of 4 and 8, respectively, probably because Fh-As_1h_ was weakly crystalline iron arsenate, whereas Fh-As_240d_ was a mixture of scorodite and hematite, which can be more easily dehydrated and polymerized into microcrystals [34].

### 3.5. Temperature Dominance

**Figure 6:**
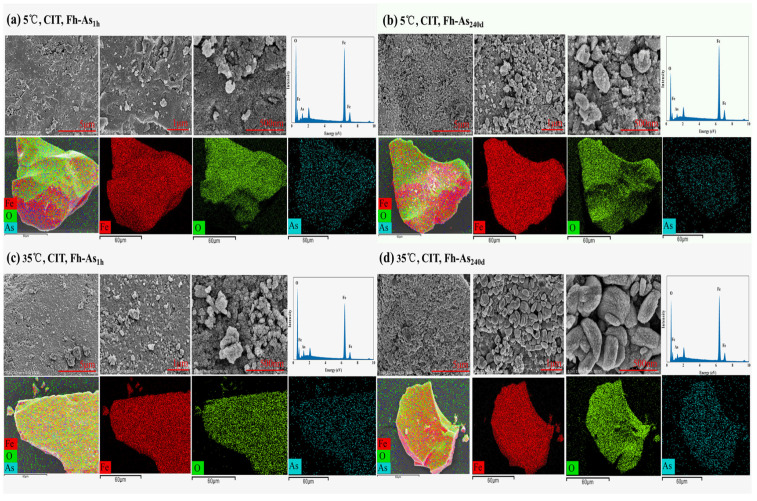
SEM images and EDS analysis of Fe-As complexes after extraction by CIT from Fh-As_1h_ and Fh-As_240d_ at 5 °C and 35 °C.

At 5 °C and 20 °C, the arsenic contents due to CIT leaching from Fh-As_1h_ were 38.67% and 29.35% lower than that due to CIT leaching at 35 °C, respectively (Figure 1). The exponential relationship between temperature and arsenic release was quantified: Release = 0.0425e^0.178(T−20)^ (R^2^ = 0.96) with 4.25% increase per 1 °C and 178% higher release at 35 °C vs. 20 °C. The intensity of the characteristic peaks of H (104) and H (110) in the XRD patterns of Fh-As_240d_ leached by PO_4_ and CIT at 35 °C were significantly stronger than those at 5 °C (Figure S3). From the SEM characterization results, as the temperature increases, the interparticle bonding becomes tighter, forming agglomerates of larger particle size. At 5 °C, 250 nm ellipsoidal particles were formed and agglomerated into minerals with smooth surfaces, whereas at 35 °C, the complexes were leached by PO_4_ to form 500 nm ellipsoidal particles and were very tightly bound into smaller, irregular blocky agglomerates with smaller pores (Figure S4), probably due to the increased temperature and the accelerated CIT-induced dissolution process of the complexes, resulting in the dissolution-polymerization of the iron oxides into new material. In particular, Fh-As_240d_ was present as irregular lumpy agglomerates after CIT leaching at 5 °C, whereas at 35 °C, elliptical particles of 300 nm were formed (Figure 6).

## 4. Discussion

### 4.1. Internal Factors Influence the Fate of As in Fe-As Complexes

The aging of ferrihydrite is the most critical internal factor affecting the stability of the complexes, which is mainly related to the different structures of the iron oxides. Ferrihydrite is a single-phase material containing 20% tetrahedral coordinated iron and 80% octahedral coordinated iron [35]. The iron atoms are present in the gaps of the faceted structure and are tightly bound together by six O+OH atoms in octahedral (γ-type) or hexahedral (α-type) forms to bring the structure into electronic equilibrium [36]. The unsaturated tetrahedra are transformation and are adsorption sites for ferrihydrite, resulting in high adsorption properties and surface activity [16]; hence, easy desorption of immobilized arsenic occurs in response to external factors [37]. In contrast, the structure of hematite consists of O^2−^ tightly stacked along the 001-crystal plane through hexagonal, with its ordered arrangement forming paired FeO_6_ octahedra. Each octahedron is cozied with three adjacent octahedra and coplanar with one adjacent octahedron; thus, its tight structure leads to an increased ability of hematite to immobilize arsenic. From a previous study, it was found that ferrihydrite contained 100% ferrihydrite at 0 d of aging, 30.39% goethite, 61.71% hematite, and 7.90% ferrihydrite in the iron oxide at 10 d of aging, 18.61% goethite, 78.83% hematite, and 2.56% ferrihydrite in the iron oxide at 30 d of aging, and 8.98% goethite, 89.66% hematite, and 1.36% ferrihydrite in the iron oxide at 60 d of aging [25]. Therefore, with increasing aging time, the content of more structurally stable crystalline iron oxides increases, leading to a decrease in the number of OH sites on the surface, prompting arsenate to bind to OH sites in the iron oxide lattice, thus forming a stable Fe-O-As bond that is less susceptible to the release of immobilized arsenic ions by the external environment [22,23]. As the aging time of the ferrihydrite increased, the content of hematite increased, and by 60 d of aging, the conversion product contained 89.66% hematite; thus, the stability of the aged mineral-As > the ferrihydrite-As complexes.

As the adsorption time increased, arsenate immobilized by the surface -OH sites of ferrihydrite gradually moved toward the mineral core and bound to the structural OH of ferrihydrite or formed complexes with new iron oxides [7], enhancing the binding capacity of iron oxides to arsenic [13], thus increasing the amount of As(V) that failed to leach. Under the influence of various environmental factors, the average amounts of arsenic released were 27.12% and 23.23% for 1 h and 240 d of reaction between Fe oxides and arsenate, respectively, and the amount of arsenic released decreased by 0.02% for each additional day within 240 d of reaction (Figure 1).

### 4.2. External Factors Influence the Fate of As in Fe-As Complexes

The stability of the Fe-As complexes was more affected by temperature than pH, i.e., for every 1 °C increase in temperature, the leached arsenic content increased by 4.25%, whereas for every 1 unit increase in pH, the leached arsenic content increased by 1.70%. This is mainly because temperature irreversibly affects the structure of FeO by reducing the active sites [10,13] at a pH of 6, as the temperature increases, FeO accelerates its conversion-crystallization process by removing OH or H_2_O [15], increasing the crystallinity of the Fe-As complexes being leached (Figure S3) and inducing the desorption release of As(V) fixed by the Fe-As complexes [13], and therefore, the stability of the bond decreases. However, the pH affects the morphological distribution of arsenic in solution and the degree of arsenate protonation on the mineral surface adsorption to change the coordinated form of arsenic on the FeO surface [29]. That is, as the pH increases, the solution proton concentration decreases and takes more protons from the FeO surface, which becomes negatively charged [8,38], leading to the repulsion and desorption of arsenic and the excitation of FeO dissolution to form irregular amorphous phases [30]. Additionally, at medium and high pH conditions, As(V) deprotonates and acts as an H-bond acceptor on the surface of ferrihydrite ore, forming a small amount of monodentate coordination [37], which is detrimental to the adsorption of arsenic by ferrihydrite ore, thereby increasing the risk of As(V) release [11]. In contrast, under acidic pH conditions, As(V) undergoes protonation or complexation on the surface of ferrihydrite and acts as an H-bond donor, forming a more stable bidentate binuclear surface complex [18]; when the H^+^ concentration is high, the mineral surface is positively charged and adsorbs H_2_PO_4_^−^ and H_2_AsO_4_^−^ under electrostatic gravity, reducing the influence of competing ions and enhancing the retention capacity of iron oxide to As(V) [21].

The average arsenic contents leached by competing anions from R1.875 and R5.66 Fe-As complexes were 41.91% or 37.26% with pH variation, while the average arsenic contents leached were 42.55% or 36.59% with temperature variation (Figure 1). The arsenic contents of PO_4_ leaching from Fh-As_1h_ complexes (R5.66) were 33.97% and 27.69% lower at pH values of 4 and 6, respectively, than that of leaching at a pH of 8, while the arsenic contents of PO_4_ leaching from Fh-As_1h_ complexes (R5.66) were lower at 5 °C and 20 °C than that of leaching at 35 °C, 49.22% and 37.41%, respectively.

According to the XPS O 1 s split-peak fit, the combined effect of pH, temperature, and competing anions affects the stability of the complexes. When extracted by PO_4_ at a pH of 8 or 5 °C, the H_2_O contents in Fh-As_1h_ were 10.43% and 12.09% (Figure 3), while those in Fh-As_240d_ were 9.79% and 11.29%. Under the influence of pH, the content of structural H_2_O increased after Fh-As_1h_ was leached by CIT, while the content of H_2_O in both Fh-As_240d_ decreased; under the influence of temperature, the content of structural H_2_O decreased after the binding was leached by CIT, and the decrease was more significant in Fh-As_240d_ than in Fh-As_1h_. When leached by CIT at a pH of 4 or 35 °C, the contents of H_2_O in Fh-As_1h_ were 11.99% and 9.15%, respectively, while the contents of H_2_O in Fh-As_240d_ were 8.23% and 8.02%. The Fe-O content of Fh-As_1h_ was 45.3%, and when leached by PO_4_ at a pH of 4 or 8, the Fe-O-As bond was broken, and the M-O (Fe-O and As-O) contents decreased to 40.92% and 37.95%, whereas when leached at 5 °C or 35 °C, the M-O contents decreased to 32.41% and 34.68%, which may be due to the elevated temperature accelerating the binding of PO_4_ to iron oxides and the replacement of PO_4_ with AsO_4_ [27,39]. The Fe-OH content of Fh-As_1h_ was 44.12%, which rose to 48.75% or 51.62% of M-OH (Fe-OH and As-OH) when leached by PO_4_ at a pH of 4 or 8. This may be due to the dissolution and release of Fe^3+^ from the iron oxide surface through the coupled dissolution and precipitation reaction mechanism of PO_4_, which supersaturates the mineral–water interface with iron phosphate and subsequently induces nucleation, agglomeration, and growth of iron phosphate nanoparticles (formation of secondary particles and laminar precipitation) [40]. In contrast, the stability of Fh-As_240d_ is affected by pH and was significantly more affected by pH than temperature: when leached by PO_4_ at a pH of 4 or 8, the M-O contents decreased to 37.93% and 36.95% (Figure 3). XPS Fe 2p, i.e., Fe 2p3/2 (711.2 eV) and Fe 2p1/2 (724.8 eV), did not shift before and after leaching, indicating that the crystallinity of the Fe oxides did not change significantly, but the Fe 2p peak areas were significantly more strongly influenced by temperature than by pH (Figure S1c,f).

### 4.3. Stability of Fe-As Complexes Under the Interaction of Internal and External Factors

The stability of the Fe-As complexes was influenced by the interaction of various factors, and the adsorption of arsenic by iron oxides was the result of a combination of electrostatic nonspecific adsorption and ligand complexation-specific adsorption [28]. At 20 °C, a pH of 4 and coexistence with citrate, Fh_60d_-As was the most stable, releasing only 10.31% arsenic; however, at a pH of 8 and coexistence with citrate phosphate, Fh_30d_-As was the least stable, releasing 49.88% arsenic. At a pH of 8, Fh_10d_-As was the least stable, releasing 41.20% arsenic. This is mainly due to the strong protonation increasing the positive charge on the iron oxide surface and strengthening the electrostatic gravity of iron oxide and H_2_AsO_4_^−^ and HAsO_4_^2−^ under acidic conditions [33], thus facilitating the fixation of arsenic. With pH, ion strength, temperature, etc., mineral surface charges accumulate, and large OH competes for adsorption sites [41,42]; thus, the electrostatic gravity gradually becomes repulsive but maintains a high adsorption capacity, and some arsenic cannot be eluted. The excitation As(V) release efficiency was relatively more stable at 20 °C with a pH of 6 and PO_4_ coupling (its slope was lower in absolute value than those at pH values of 4 and 8) (Figure S6a), whereas the excitation As(V) release efficiency was higher with increasing aging time at a pH of 6 and CIT coupling than at pH values of 4 and 8 (its slope was greater in absolute value than at pH values of 4 and 8) (Figure S6b).

The stability of the complexes decreases under the coupling effect of increasing temperature and decreasing ferrihydrite aging time, while CIT inhibits the re-adsorption of arsenic to the mineral surface [9]. The efficiency of excitation of As(V) release with increasing aging time at 35 °C is relatively higher than those at temperatures of 5 °C and 20 °C (the absolute values of their slopes are greater than those at 5 and 20 °C) (Figure S7), indicating that the process is influenced by the interaction of temperature and ferrihydrite aging. Based on the previous research results, we developed a quantitative stability prediction model: Stability Index (SI) = (Fe/As Molar Ratio × Aging Time (days))/T^1.2^ (R^2^ = 0.91), introduced temperature sensitivity coefficient (4.25%/°C) for aquifer systems. During the reaction, the Fe-As complexes surface active site or pore is saturated for oxalate accumulation, causing a certain amount of citrate to not directly bind to the mineral surface but form an organic acid polymolecule layer on the mineral surface, thus, covering the dissolution site [21], making the arsenic release and absorption limited by steric hindrance. Fh_60d_-As was most stable at a pH of 6 and 5 °C with citrate, releasing only 10.15% arsenic; Fh-As was least stable at 35 °C with citrate, releasing 59.82% arsenic. But the current model and its validation were primarily based on a laboratory system. Its core value lied in quantitatively revealing the relative importance of controlling factors (sensitivity analysis) and providing a verifiable quantitative relationship. This offers key mechanistic support and a preliminary framework for risk assessment in field applications.

With the interaction of various internal and external factors, temperature is the most critical factor affecting the stability of the complexes. At 20 °C and a pH of 4, Fh_60d_-As was the most stable, releasing only an average of 18.83% arsenic, followed by the next most stable at 5 °C and a pH of 6, releasing only an average of 19.07% arsenic. At 35 °C and a pH of 6, Fh-As was the least stable, releasing an average of 52.39% arsenic, followed by Fh_10d_-As at 35 °C and a pH of 6, which was the next least stable, releasing an average of 48.12% arsenic. For field application, maintaining temperatures below 25 °C could be achieved through engineering strategies such as insulating cover layers, reflective surfaces, or subsurface placement to leverage soil thermal inertia. Where full temperature control is impractical, prioritizing the use of pre-aged (e.g., 60 d) iron-based materials becomes paramount to mitigate temperature-driven arsenic release risks.

It should be noted that citrate was selected in this study as a model organic ligand to elucidate a specific competitive mechanism—namely, the ligand-promoted dissolution induced by low-molecular-weight organic acids. However, natural organic matter (NOM) in real-world environments, such as humic and fulvic acids, exhibits far more complex composition and behavior. NOM contains abundant functional groups (e.g., carboxyl and phenolic hydroxyl groups) and covers a broad molecular-weight distribution, which can influence Fe–As complexes stability through multiple pathways: (1) NOM can directly compete with arsenate for specific adsorption sites on iron oxide surfaces; (2) NOM may act as a bridge, participating in the formation of Fe(III)–NOM–As(V) ternary surface complexes, thereby altering the speciation and mobility of arsenic; and (3) adsorption of NOM onto mineral surfaces can slow or even hinder the transformation of ferrihydrite into more thermodynamically stable crystalline phases (e.g., goethite, hematite), consequently affecting the long-term arsenic-stabilization capacity [17,18]. Therefore, although the model system used here reveals fundamental principles governing competitive ion effects, the site-specific composition, concentration, and resultant complex interfacial reactions of NOM must be taken into account when assessing actual field risks or designing remediation strategies. Future studies should systematically investigate the quantitative impact of NOM from different sources and across various molecular-weight fractions on Fe–As complexes stability, and incorporate NOM parameters into predictive modeling frameworks, thereby bridging the gap between laboratory-based mechanistic understanding and field-scale environmental management.

## 5. Conclusions

This study systematically investigated the impacts of environmental factors and material properties on the stability of Fe-As complexes and developed a long-term stability model for iron-arsenic compounds. Our study established three key findings: (1) temperature emerged as the dominant factor regulating arsenic release (4.25% increase per 1 °C), with its impact surpassing that of pH (1.70% increase per pH unit) and competitive anions; (2) ferrihydrite aging enhanced Fe-As complex stability via phase transformation to hematite (89.66% hematite content at 60 d of aging), accompanied by a 1.2 eV rise in As(V)-O-Fe binding energy, reducing arsenic release to 18.83% under pH 4/20 °C conditions; (3) phosphate induced 2.4-fold higher arsenic release than citrate, while acidic conditions (pH 4–6) suppressed arsenic mobilization, and the developed stability prediction model (R^2^ = 0.91) enabled site-specific arsenic toxicity risk assessment.

For practical arsenic pollution remediation and toxicity risk control, we recommend: (1) maintaining arsenic-containing waste repository temperatures below 25 °C to mitigate temperature-driven release; (2) utilizing 60 d pre-aged iron-based materials to enhance the long-term stability of remediation systems; (3) considering competitive anion speciation (e.g., phosphate-rich vs. citrate-rich scenarios) in site-specific risk evaluations. Note that this study did not account for the effects of soil organic matter or redox potential, which warrants further investigation for comprehensive toxic metalloid management.

## Data Availability

The original contributions presented in this study are included in the article/Supplementary Material. Further inquiries can be directed to the corresponding authors.

## Supplementary Materials

The following supporting information can be downloaded at: https://www.mdpi.com/article/10.3390/toxics14010104/s1, Figure S1. XPS scanning spectra of Fe-As complexes before and after extraction by PO4 and CIT at pH values of 4, 6, and 8 (a) or at 5 °C, 20 °C, and 35 °C (d). As 3d scanning spectra of Fe-As complexes before and after extraction by PO4 and CIT at pH values of 4, 6, and 8 (b) or at 5 °C, 20 °C, and 35 °C (e). Fe 2p scanning spectra of Fe-As complexes before and after extraction by PO4 and CIT at pH values of 4, 6, and 8 (c) or at 5 °C, 20 °C, and 35 °C (f); Figure S2. SEM images and EDS analysis of Fh-As1h (A) and Fh-As240d (B); Figure S3. XRD pattern of Fe-As with Fe/As molar ratios of (A) R5.66 and (B) R1.875 before and after extraction by PO4 and CIT at 5 °C, 20 °C and 35 °C, respectively. The newly formed substances are indicated in the graph as follows: H: hematite; Figure S4. SEM images and EDS analysis of Fe-As complexes after extraction by PO4 from Fh-As1h and Fh-As240d at 5 °C and 35 °C; Figure S5. SEM images and EDS analysis of Fe-As complexes after extraction by CIT from Fh-As1h and Fh-As240d at pH values of 4 and 8; Figure S6. The correlation between the ratio of the As(V) content extracted from the Fe-As complexes by PO4 (a) and CIT (b) to the total adsorbed arsenic and the aging of ferrihydrite at 20 °C and different pH values; Figure S7. The correlation between the ratio of the As(V) content extracted from the Fe-As complexes by PO4 (a) and CIT (b) to the total adsorbed arsenic and the aging of ferrihydrite at a pH of 6 and different temperatures.
