# Supplementary material for: Mechanisms Governing the Stability of Fe-As Complexes: Roles of Environmental and Material Intrinsic Factors
Source: Toxics. 2026 Jan 22;14(1):104. doi: 10.3390/toxics14010104 (PMC12846290; doi:10.3390/toxics14010104)
Supplement: Supplementary file 1 [file toxics-14-00104-s001.zip › toxics-4070989-supplementary.pdf]

toxics

Supplementary data

Mechanisms Governing the Stability of Fe-As Complexes: Roles of Environmental and Material Intrinsic Factors

Zhonglan Yang [1,2], Tianlai Ouyang [1], Shiming Su [2], Yanan Wang [2], Fengxian Yao [1], Zhiqiang Ding [1], Mengmeng Yan [1,*] and Xibai Zeng [2,*]

[1] National Navel Orange Engineering Research Center/School of Life Sciences, Gannan Normal University, Ganzhou 341000, China; zhonglanyang@163.com (Z.Y.); 18574367330@163.com (T.O.); fengxianyao@aliyun.com (F.Y.); zhiqiangding1997@outlook.com (Z.D.)

[2] Institute of Agricultural Environment and Sustainable Development, Chinese Academy of Agriculture
Sciences, Beijing 100081, China; sushiming@caas.cn (S.S.); wangyanan@caas.cn (Y.W.)

* Correspondence: yanmengmeng2021@163.com (M.Y.); zengxibai@caas.cn (X.Z.)

Figure

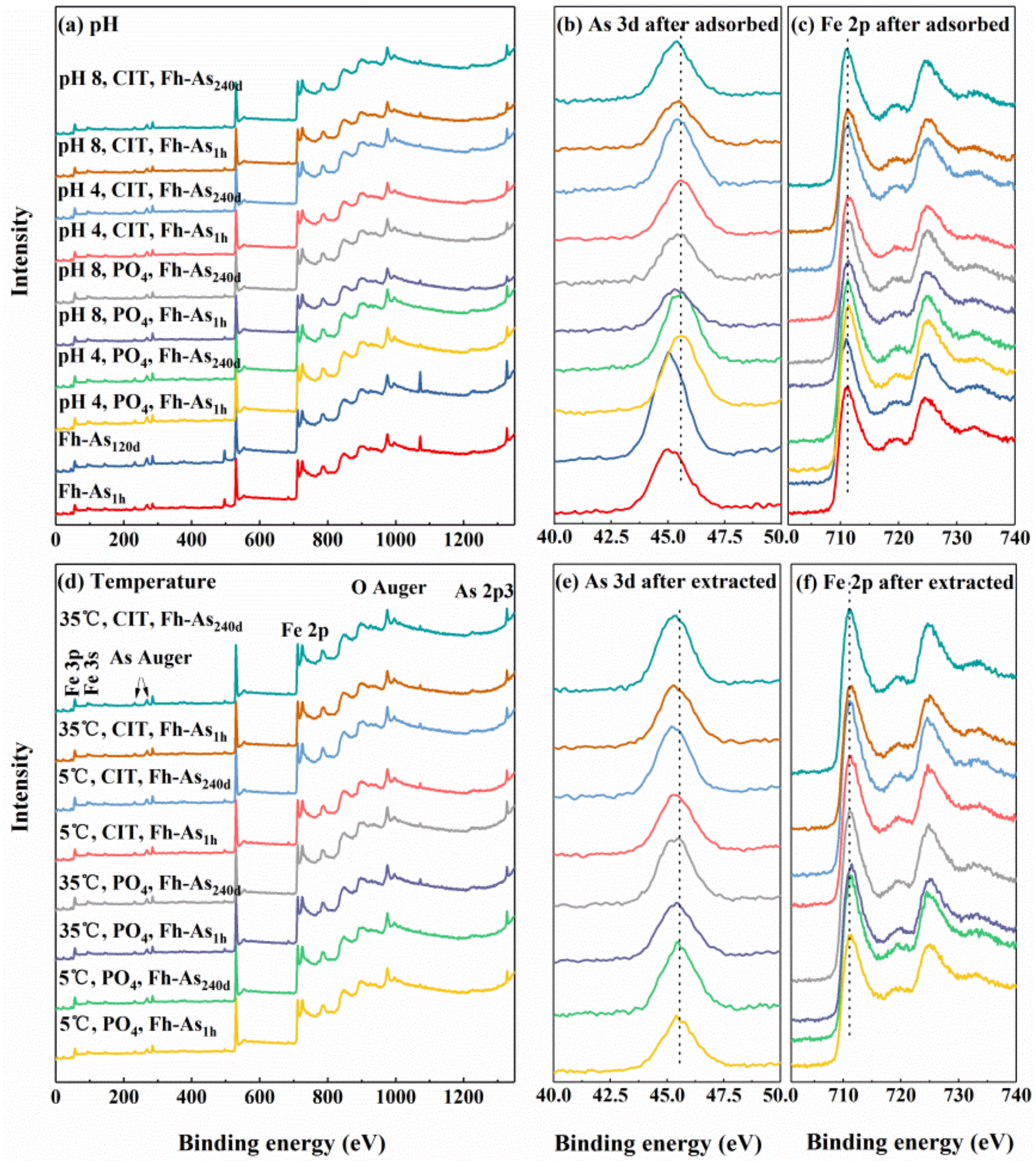

Figure S1 XPS scanning spectra of Fe-As complexes before and after extraction by PO_4 and CIT at pH values of 4, 6 and 8 (a) or at 5 °C, 20 °C and 35 °C (d). As 3d scanning spectra of Fe-As complexes before and after extraction by PO_4 and CIT at pH values of 4, 6 and 8 (b) or at 5 °C, 20 °C and 35 °C (e). Fe 2p scanning spectra of Fe-As complexes before and after extraction by PO_4 and CIT at pH values of 4, 6 and 8 (c) or at 5 °C, 20 °C and 35 °C (f).

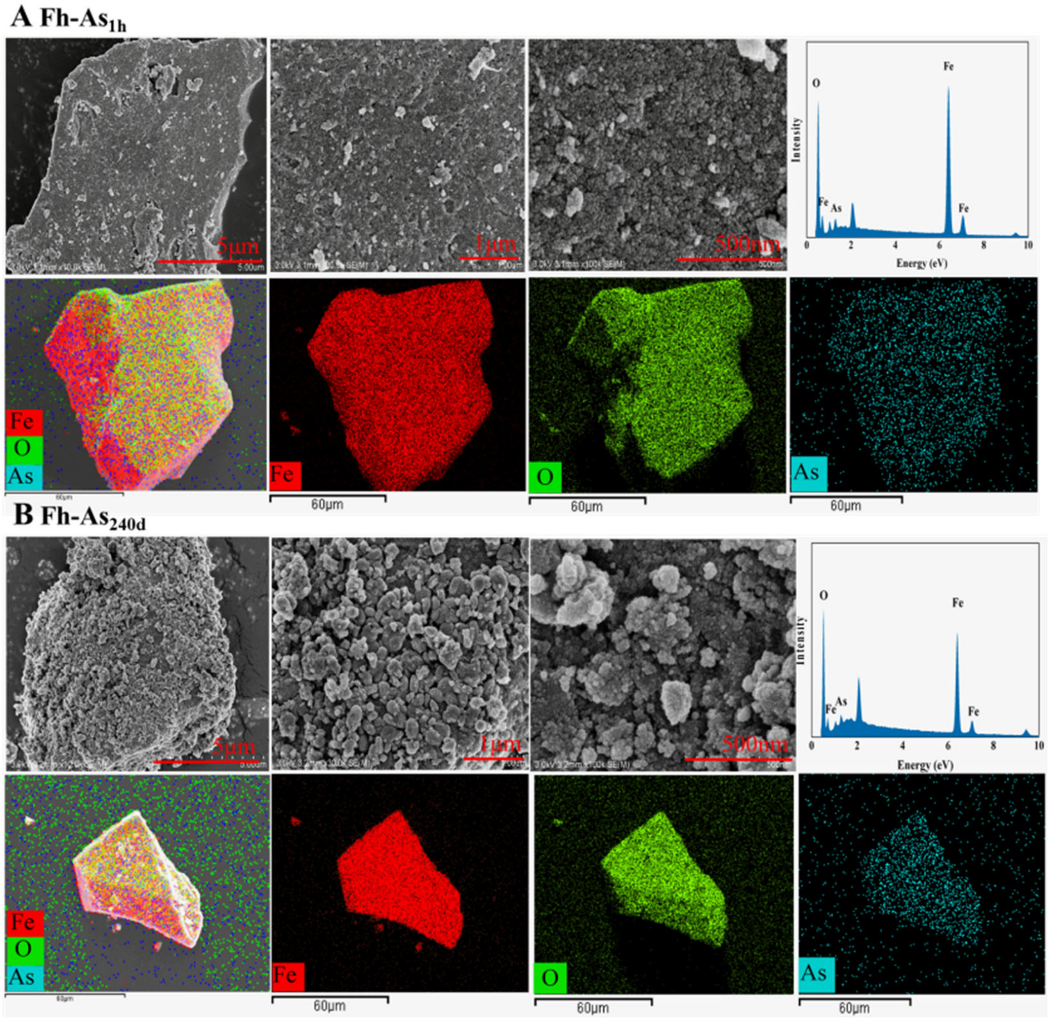

Figure S2 SEM images and EDS analysis of Fh-As_{1h} (A) and Fh-As_{240d} (B).

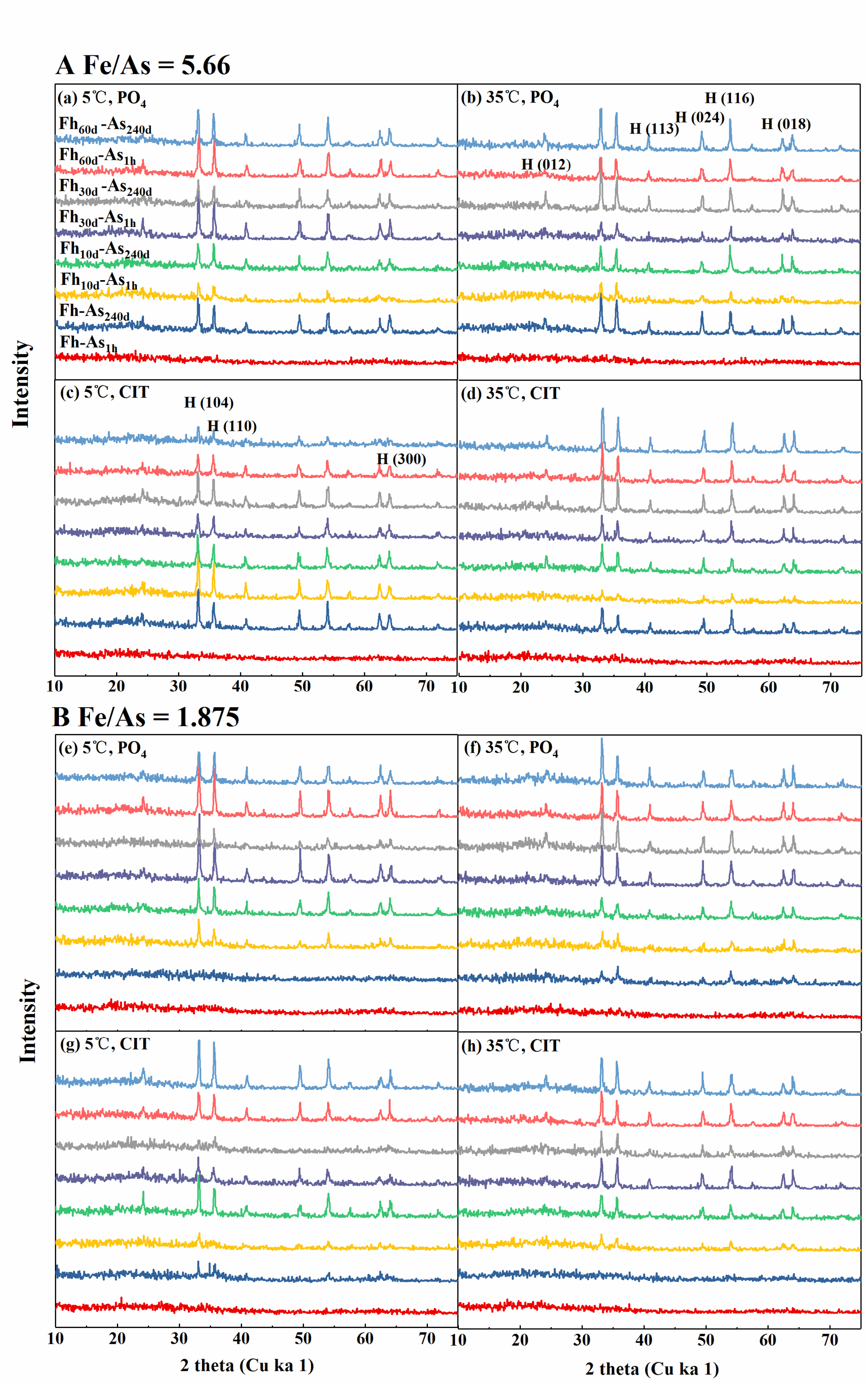

Figure S3 XRD pattern of Fe-As with Fe/As molar ratios of (A) R5.66 and (B) R1.875 before and after extraction by PO_4 and CIT at 5 °C, 20 °C and 35 °C, respectively. The newly formed substances are indicated in the graph as follows: H:

hematite.

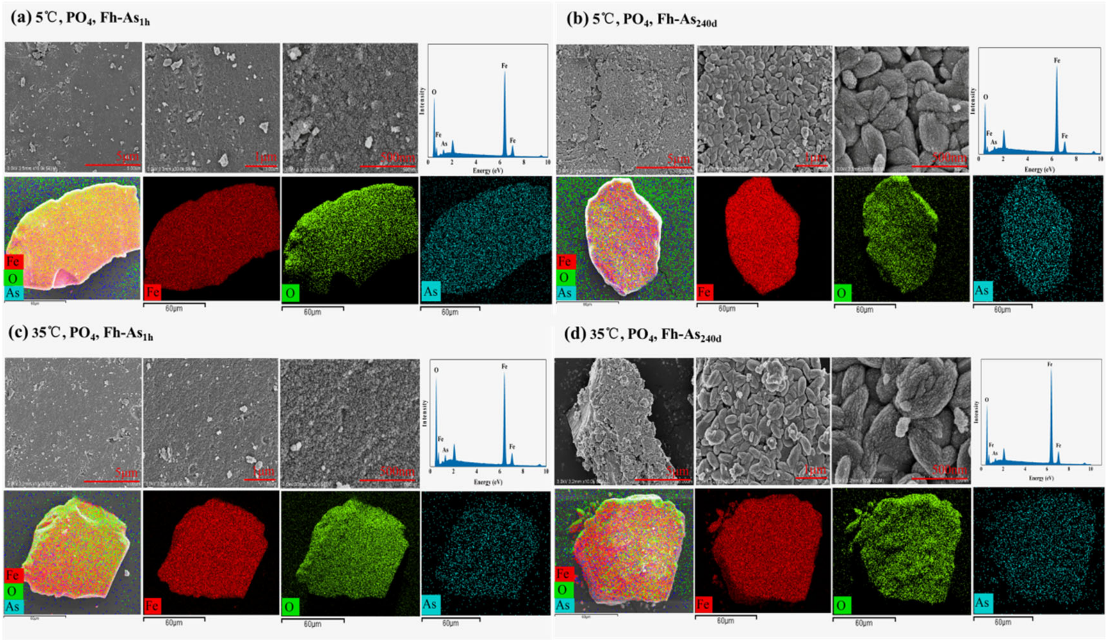

Figure S4 SEM images and EDS analysis of Fe-As complexes after extraction by PO_4 from Fh-As_{1h} and Fh-As_{240d} at 5 °C and 35 °C.

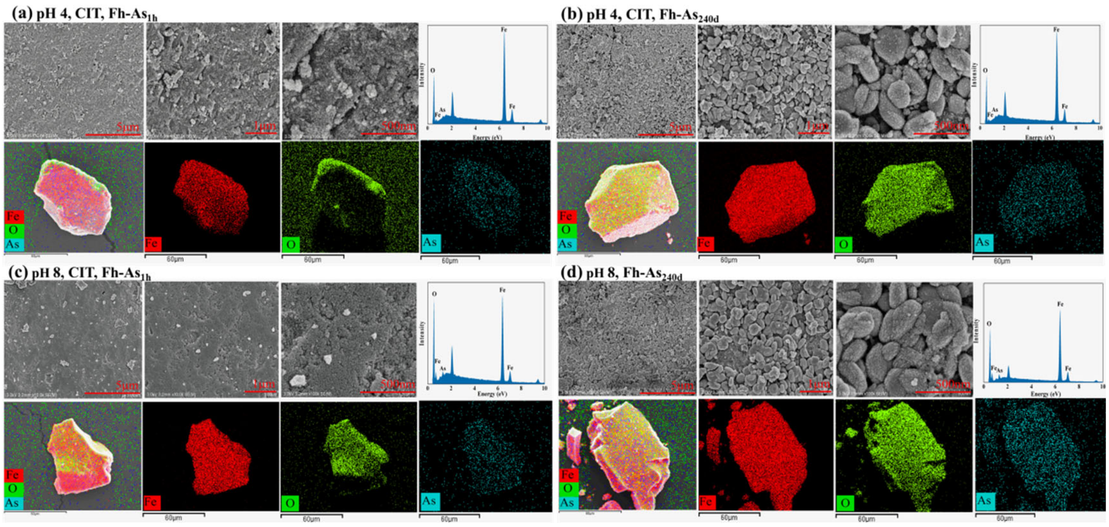

Figure S5 SEM images and EDS analysis of Fe-As complexes after extraction by CIT from Fh-As_{1h} and Fh-As_{240d} at pH values of 4 and 8.

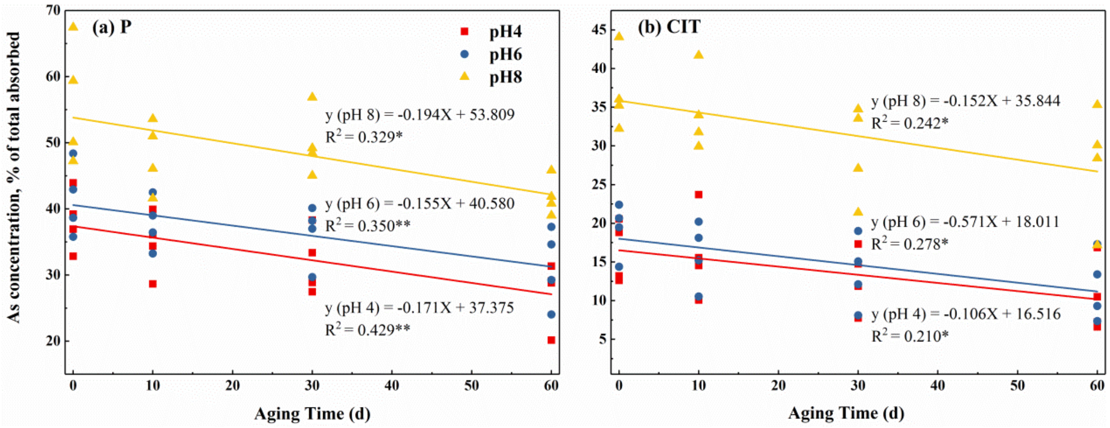

Figure S6 The correlation between the ratio of the As(V) content extracted from the Fe-As complexes by PO_4 (a) and CIT (b) to the total adsorbed arsenic and the aging of ferrihydrite at 20 °C and different pH values.

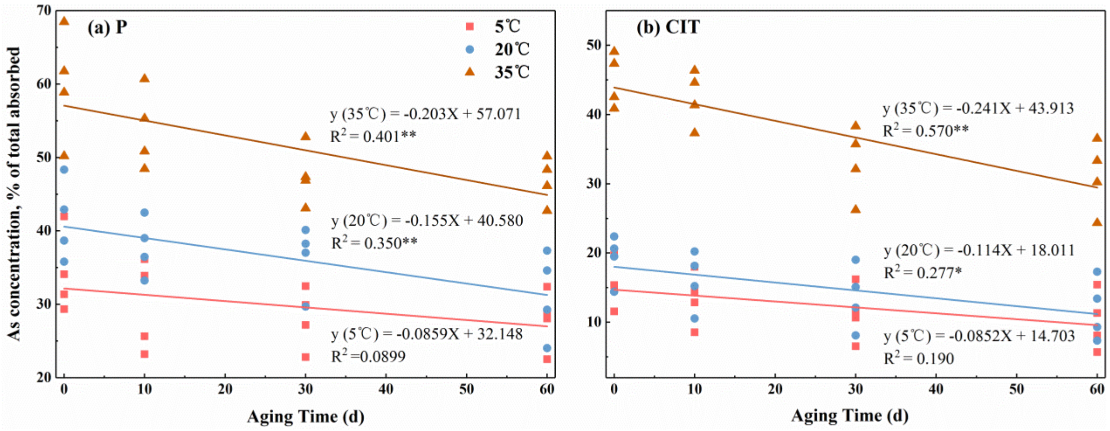

Figure S7 The correlation between the ratio of the As(V) content extracted from the Fe-As complexes by PO_4 (a) and CIT (b) to the total adsorbed arsenic and the aging of ferrihydrite at a pH of 6 and different temperatures.
